# Suppression of erythropoiesis by *Plasmodium vivax* infection

**DOI:** 10.1186/1475-2875-9-S2-P35

**Published:** 2010-10-20

**Authors:** Tasanee Panichakul, Witchuda Payuhakrit, Chokdee Wongborisuth, Suradej Hongeng, Rachanee Udomsangpetch

**Affiliations:** 1 Faculty of Science and Technology, Suan Dusit Rajabhat University, Bangkok, Thailand; 2 Department of Pathobiology, Faculty of Science, Mahidol University, Bangkok,Thailand; 3 Research Center, Faculty of Medicine, Ramathibodi Hospital, Mahidol University, Bangkok, Thailand; 4 Department of Pediatric, Faculty of Medicine, Ramathibodi Hospital, Mahidol University, Bangkok, Thailand

## Background

**Figure 1:**
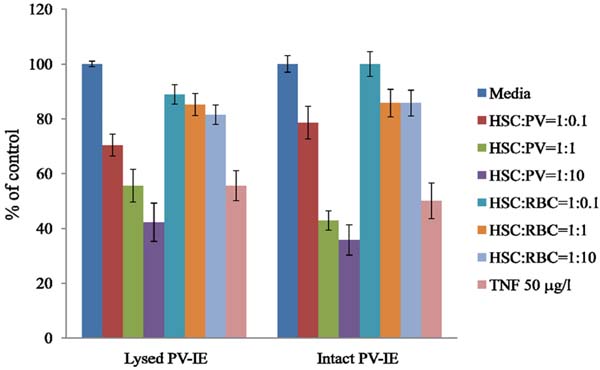
Inhibition of erythroid expansion. HSCs, 5 day-old, 10^5^ cells were cultured with intact or lysed PV-IE and further cultured up to 12 days. Intact and lysed RBCs and TNF-α were as controls.

Reports of severe anemia due to *P. vivax* infection increase [1]. *P. vivax* is considered to infect reticulocytes and parasitemia is generally low, this suggests that in addition to the simple destruction of infected red cells there is another mechanism to induce anemia in vivax. A report of vivax malaria in bone marrow from severely anemia patients exhibited dyserythropoiesis [2]. Our study demonstrated that *P. vivax* could infect erythroblasts and erythroblast loss was at least partially attributed to direct killing by parasite invasion [3]. However, the mechanism involving in induction of anemia in vivax malaria is still unclear. Here, hematopoietic stem cells (HSCs)/CD34^+^ cells from normal human cord blood were subjected to study the suppression of erythropoiesis by *P. vivax* infection. Erythroid cells derived from HSCs were cultured in serum-free medium supplemented with growth factors and cytokines. Intact or lysed *P. vivax*-infected erythrocytes (PV-IE) isolated from patient blood were added to erythroid cultures. Results showed both intact and lysed PV-IE could inhibit erythroid expansion by up to 50-55 % compared with controls containing red cells (RBCs) (see Figure 1).

The reduction of erythroid expansion was not significantly greater by intact PV-IE when compared with lysed PV-IE. The inhibition of cell expressing glyphorin A was up to 66.67 % compared with controls.

## Conclusion

*P. vivax* could inhibit erythroid growth and development and this suppression of erythropoiesis by *P. vivax* infection is plausible to involve in induction of anemia.
